# Public educational psychology services in Israel on the internet

**DOI:** 10.1186/s13584-019-0298-4

**Published:** 2019-03-18

**Authors:** Sarit Alkalay, Avivit Dolev

**Affiliations:** 1 Department of Psychology, Max Stern Jezreel Valley College, 1930600 Emek Jezreel, Israel; 2 Technion (Israel Institute of Technology), Faculty of Education in Science and Technology, Haifa, Israel

**Keywords:** Public Educational Psychology services, Children and youth, Internet site, Mental health

## Abstract

**Background:**

The public Educational Psychology Services provide mental health services for children and youth in Israel, alongside the Ministry of Health and the Ministry of Social Affairs and Services. The Psychological and Counseling Services Division of the Ministry of Education (known as SHEFI - Sherut Psychology Yeutzi), funds and supervises local Educational Psychology Services which are aimed at supporting child development and enhancing the emotional welfare of children and their families. The demand for the services of educational psychologists is increasing. Yet this demand is not being met due to the insufficient number of job slots allocated, the geographical distances in outlying peripheral areas, the already high loads in the psychologists’ daily routine, and other such problems. A wide range of effective psychological services can be offered via the internet. The internet therefore has the potential to serve as a useful and efficient missing link between the high demands for educational psychology services on the one hand and the ability and desire among educational psychologists to meet those needs on the other. Moreover, even if the services were fully staffed, the resources would still be insufficient to provide personal (face-to-face) treatment for all, so that internet-based access to services would still need to be developed. Those services provide unique advantages such as overcoming distance and enabling higher availability of mental health professionals. The objectives of the current study were to describe the prevalence of public educational psychology services available online in Israel, with specific focus on the Arab minority and the peripheral regions, and to highlight the benefits of expanding those services.

**Method:**

During 2016, we conducted a survey comprising all 252 Public Educational Psychology Service units in Israel (*n* = 170 in the Jewish sector, and *n* = 82 in the Arab sector). The method used to search for online sites was in line with the actions taken by an average end-user searching for information on the internet.

**Results:**

The survey found that 125 of the units in the Jewish sector (73.5% of those units) and all 82 units in the Arab sector had no online site at all, constituting 82.2% of all the units in Israel. Of the 45 Jewish websites located by the survey, 42 (93.3% of the sites) were not user friendly (not interactive), and only three offered the possibility of interacting with psychologists (6.7% of the sites). Nevertheless, all the sites (*n* = 45) offered a high degree of quality and variety that exceeded basic information.

**Conclusion:**

We believe that the presence of educational psychologists on the internet is essential in order to meet the challenges presented by the growing needs of students, parents and teachers in the current digital era. The survey revealed that the public educational psychology system in Israel has not yet bridged the technological gap. Special attention should be directed to the peripheral regions and to the Arab sector, where the technological services can make a significant contribution. The local public services’ attempts to create and operate websites (45 Jewish websites according to the survey), are indicative of the determination to offer psychological support to the community at large, and of the ambition to overcome availability and accessibility problems. The concept of internet services might be useful not only for the SHEFI, but also for the array of mental health services for children and youth in Israel. Thus, we recommend that a policy should be formulated regarding internet-based mental health services for children and youth in Israel, and we call for a collaboration between the various ministries in implementing this process.

## Background

For over five decades, the public Educational Psychology Services in Israel have been working to support child development and enhance the emotional welfare of children and their families [1, 2]. In so doing they are mandated by regulations reflecting the Ministry of Education’s resolve to prioritize children’s well-being [3]. Over the years, the professional psychologists provided real added value and made a genuine contribution to the well-being of children and adolescents, as can be seen in the growing demands for psychology services in the schools [4]. Continuous efforts to supply these services have encountered a major deficiency that in recent years threatens to lower dramatically the ability of the public services to fulfill their duty. In 2007, the State Comptroller declared that in close to half of Israel’s local authorities (municipal councils, local councils) only 50% or fewer of the actual job slots for psychologists were filled, and in approximately another quarter of the authorities the percentage of filled positions was even lower than 30% [5]. In 2013 the Chief Psychologist of the Israel Ministry of Education (MOE) stated that only 65% of the required psychologist positions were filled [6]. By 2017, the Chief Psychologist reported that only 68% of the slotted positions were filled. The report also mentioned that the situation regarding the number of positions for psychologists and the ability to fill them is better in the Jewish sector than in the Arabic sector [7].

Technology can help in realizing the goodwill and expertise of the public Educational Psychology Services and meeting the needs of Israeli school pupils and their families by overcoming the persistent discrepancy between the allocated number of psychologist positions and the actual number of psychologists. Additionally, even if the services were fully staffed, the resources would still be insufficient to provide personal (face-to-face) treatment for all, so that internet-based access to services would still need to be developed. Among the reasons for developing such online psychological services are to provide greater access to services, to offer flexible availability [8], to reduce the stigma of using such services [9], and to reach population groups that might tend to avoid face-to-face encounters [10, 11]. Based on a growing number of studies showing the efficacy of online psychological services (e.g. [12]), we assume that the internet has major potential to promote the objectives of the Educational Psychology Services and to assist in providing psychological support to the population at large. The results of our modest experience in establishing a website for the public Educational Psychology Services unit in Beit Shean during 2010–2014 were encouraging. Hence, we were motivated to examine the overall situation of online psychological services in Israel and to promote the potential for such online services. The goal of the current study was to describe the prevalence and nature of Israeli public Educational Psychology Services online by examining the status of their websites.

The Israel MOE defined children, their parents, and the educational staff working with them as the clients of the Public Educational Psychology Services [3]. These clients now live in the digital era of the twenty-first century in which the internet dominates the public space. More than 7.2 billion people (around 39% of the world’s population) make frequent use of the internet, with the rate of worldwide internet use increasing by 570% during the last decade [13]. Alongside this consistent rise in the number of adult internet users, it is evident that particularly among the younger generation the online future is already here. In Israel young people spend an average of 2–4 h per day surfing the net [14]. More than 1,350,000 Israeli children and adolescents up to the age of 18 have internet access and surf the net, constituting 94% of this age group [13]. It is only natural for adults and children alike to look for psychological information and psychological services online, just as they look for other services online (e.g., services from government agencies).

### Psychological services online

Telepsychology or tele-mental health services are defined as all interactions between healthcare professionals and their patients that are not in person [15]. A wide range of psychological services can be offered via the internet, ranging from general support to intensive therapy. Professional information can be posted on the internet, thus enabling users to help themselves. Client-psychologist communication via email or on designated forums allows users to consult occasionally with a professional. Clients that are engaged in psychological therapy can use the internet as another channel that can replace the traditional face-to-face interaction or enhance it by means of video sessions and ongoing text chats between meetings. Finally, various applications can be used in therapy to treat specific conditions (e.g. anxiety, PTSD, social skills) [16].

Successful attempts are already being made to use the internet in the field of mental health for wide scale consultation and for personal therapy. A growing body of evidence suggests that online treatments, among them internet-CBT for anxiety and depressive disorders, integrate traditional components of the therapeutic relationship [17, 18] and are cost-effective, practical and often result in outcomes similar to those of face-to-face treatments [10, 19, 20]. The success of online treatments is a promising sign for the effectiveness of internet interventions on the part of educational psychologists. Educational psychology interventions entail some practices that resemble those used in online psychological therapy (e.g., reflection, mirroring, interpretations). Yet unlike psychological therapy, the educational psychology interventions are considered to be more focused, supportive, short-term actions aimed at quickly and efficiently tackling a current problem and assisting the individual in regaining balance and calmness [1, 2]. In fact, the literature also includes examples of this kind of online psychological intervention. For example, Hanley & D'Arcy [21] reported on an organization in Britain (The Samaritan) that offers psychological support to children and adolescents over the internet. The users can send an email and consult with a professional whenever they need, without having to identify themselves or to engage in ongoing psychological therapy. The Samaritan email support system received and answered 36,500 queries in 2000, 72,000 queries in 2002 and 184,000 queries in 2006.

Nevertheless, it should be noted that despite the many similarities between online and face-to-face (FTF) treatments and despite the unique advantages of online psychological interventions, online interventions are not suitable for all users and may also have some negative effects. According to Rozental et al. [20], compared to FTF treatments, internet psychological interventions involve a lower level of control. Moreover, communication is lacking non-verbal stimuli which may lead to misinterpretations of significant therapeutic signals. Additionally, unguided self-help may be related to a greater risk of misunderstanding the treatment rationale [20].

### The significance of online psychological services for the Arab minority population and for the peripheral regions in Israel

The option of seeking help online may be particularly important for specific at-risk populations. Amichai-Haburger, Brustein Klomek, Friedman, Zuckerman, & Shani-Sherman [9] suggested that it may be easier for some people to undergo online treatment rather than traditional face-to-face treatment because online treatment is likely to have less of a stigma associated with it. In line with this assumption, Khan (Khan MA: Exploring black, Asian and minority ethnic young people’s attitudes towards accessing online and face-to-face counselling, unpublished) reported that due to stigma, minority youth (Black, Asian and minority ethnic) in the UK showed a preference for this help-seeking media over FTF counseling. Consequently, we were specifically interested in the online accessibility of public Educational Psychology Services in Israel to the Arab minority in Israel.

Of the 8,793,000 residents of Israel, most are Jewish (74.6%), while 20.9% are Arab (Muslims, Christians, and Druze), and 4.5% are from other groups [22]. Among the young members of the Arab minority in Israel, an insufficient number utilize mental health services. A study conducted in Israel found that 10–12% of those in the youth sample met the DSM criteria for mental disorders [23]. Nevertheless, a large proportion of needs are unmet among all participants, and especially among the Arab minority youth. Of those who met the criteria for mental disorder, 54% of the Jewish sample and 91% of the Arab minority sample had not sought help [24]. The reasons for this sharp contrast between the Jewish and Arab sectors may reflect the reasons Khan (Khan MA: Exploring black, Asian and minority ethnic young people’s attitudes towards accessing online and face-to-face counselling, unpublished) found in his study of minorities in Britain, namely the stigmas associated with mental health problems within their communities. The use of the internet for providing psychological services may offer a way to overcome this obstacle.

Technology helps bridge geographical distances and make services available even when they are not physically accessible [8]. In Israel, the peripheral regions primarily constitute the northern and southern regions. Approximately a quarter of the population (roughly 2.5 million people) live in those regions (roughly 1.3 million in the north and 1.1 million in the south), in an area spread over 75% of Israeli terrain (20.9% in the north and 64.9% in the south). More than half of the localities in Israel are located in those regions (*n* = 666) [25]. These statistics indicate that the localities in the peripheral areas are small (sometimes with a population as low as several hundred) and are spread over a large territory. The sparseness of the population and its relative isolation from the center of Israel make medical and mental health services less available and accessible.

There is consensus among mental health professionals and policy -makers in Israel regarding a shortage of professionals in the periphery and of professionals sufficiently attuned to the unique needs of the Arab and ultra-Orthodox populations [26]. Thus, the internet may play a crucial role in the attempt to provide services to the peripheral areas and to those populations.

### Overview of public mental health services in Israel

In 2013, a review of child and adolescent mental health (CAMH) services in Israel was published [26]. One of the major issues the report addressed was coping with behavioral problems and mental illness (hyperkinetic problems, emotional problems, conduct problems, psychotic problems) among children and youth (birth-18 years) as defined by DSM-IV-R. The report mapped the various organization that are responsible for providing these services. At the time of the report, the Israeli Ministry of Health (MOH) was the governmental agency responsible for developing and coordinating mental health services. The MOH operated 38 CAMH community clinics (“tachanot yeledim v’noar”) throughout Israel, with many clustered in the center of Israel. Typically, these services are managed by clinical psychologists or social workers and employ a range of staff including clinical psychologists, social workers, expressive arts therapists and occupational therapists. The resources at most of the community clinics are limited. These clinics provide some assessment of mental health problems, but this does not include any cognitive or neuro-psychometric component. It was stated that the largest health plan in Israel, “Clalit”, on behalf of the MOH, operates 18 community clinics, as well as a number of hospital-based clinics, mainly in the center of the country. According to the report, many families had to wait several months before an initial assessment, and several additional months before receiving treatment. Most interventions in the MOH and health plan clinics have a psychodynamic orientation, and there are virtually no cognitive-behavioral therapy interventions. The typical treatments are focused on the individual child and last 12 or more months. All services provided by the clinics are free with no co-payment. Also, the 2013 report specified that most of the large hospitals in Israel have inpatient or outpatient clinics providing CAMH services. These services are managed by psychiatrists, and typically have staff teams that comprise of clinical psychologists, nurses (inpatient wards), social workers, and occasionally, occupational therapists. Many outpatient clinics have 1–2 month waiting times for initial assessment and then a much longer waiting time for receiving therapeutic treatment.

In 2015 the Israeli government has launched an extensive reform in mental health services, assigning responsibility for providing those services to the four major health-plans [27, 28]. The reform was initiated in order to strengthen the connection between physical health services and mental health services. Additionally, the reform intended to facilitate accessible and available services, to improve the quality of mental health care, to reduce the stigma associated with seeking and receiving mental health services, and to improve the functioning of public health systems [27, 28]. Thus, all the Israeli health plans now have extensive networks of community-based mental health services for children involving psychiatrists, psychologists, social workers, primary care physicians, and others. Following the 2015 reform, more people are seeking and receiving public mental health services. A year and a half after the initiation of the reform, an increase in referrals was registered, with 30,000 new patients seeking mental health services, approximately 22% of them (*n* = 6600) were children [29]. Additionally, the health plans are now making efforts to culturally adjust the services that are provided to Arabs, the ultra-orthodox Jews (Haredim), and to other groups with unique needs [28]. Alongside the improvements that are associated with the reform, there are lingering complications in providing mental health services in Israel. For example, the Ministry of Health has allocated funds to the health plan for employing clinical psychologists. However, there are not enough experts in clinical psychology in Israel. Also, the health plans have not utilized the governmental funding solely for positions designated for clinical psychologists. Thus, a shortage in manpower has resulted in the endurance of long waiting lists [29, 30].

The Ministry of Education (MOE) via the public Educational Psychology Services (see the next section regarding services provided by the MOE) and the Ministry of Social Affairs and Services (MOSAS) are also involved in providing some mental health and related services to children and youth. Accordingly, the degree of coordination among the three ministries around mental health care issues can have an important impact on the extent to which mental health needs are met effectively [26]. A successful example of cooperation and coordination between the ministries, is the National Program for Suicide Prevention [31]. The program was initiated and funded by the MOH. The program includes, among other things, training of educational psychologists to provide risk assessments of suicide threats made by children and youth. Following the initial assessment, educational psychologists continue to treat the children, in collaboration with their families and the educational staff. Since 2015 educational psychologists conducted more than 2700 risk assessments and provided therapy to more than 800 children and youth that were considered to exhibit suicide risk [32].

### Overview of public Educational Psychology Services in Israel

Educational psychologists in Israel are licensed by the MOH. As part of the licensing process they are required to do an extensive internship and pass a qualification exam [33, 34]. In contrast to many school and educational psychology services around the world, which tend to focus on learning problems and school behaviors, educational psychologists in Israel also have an important community-based role in providing some mental health services [26]. The designated tasks entrusted to educational psychologists working with children and adolescents in Israel include diagnosing and treating learning disabilities, attention deficit disorder, intellectual disabilities, conduct disorder, and emotional disturbance [35]. Such diagnoses can also be made by other mental health professionals (e.g., clinical or developmental psychologists, or psychiatrists). However, because educational psychologists are deployed in all the schools and kindergartens in Israel, most diagnoses of children and adolescents are likely to be made by them. Educational psychology interventions are considered to be more focused, supportive, short-term actions aimed at quickly and efficiently tackling a current problem and assisting the child in regaining balance and calm. Those interventions often involve parents and educational staff [1, 2]. Thus, in some aspects of their professional duties, educational psychologists are working in a similar fashion to other mental health professionals, and in some aspects educational psychologists’ professional characteristics also include unique psychological interventions that do not adhere to the standard model of psychological treatment.

The Psychological and Counseling Services Division of the MOE (known by the acronym SHEFI - Sherut Psychology Yeutzi) has been entrusted with promoting the well-being and emotional health of all pupils in the educational system [1, 36]. The local Educational Psychology Services units meet this objective by working within the educational systems (schools and kindergartens) and with the community at large, as well as with individuals and families during routine times and emergencies. The services are intended for students, parents, the educational staff in the kindergartens and schools and all community organizations that work with this population [3, 5]. The MOE and the local government authorities fund approximately 2800 positions for educational psychologists [4]. These psychologists are employed through the local municipalities via public Educational Psychology Service units, with one educational psychologist position allocated for approximately one thousand children [3]. There is a close working relationship between SHEFI and each local unit in terms of policy development, procedural guidelines for dealing with a variety of children’s problems, and supervision of the overall system. SHEFI also provides continuing training for the educational psychologists, as well as additional funding for specific innovative pilot projects [26].

The routine work of educational psychologists is varied and complex and includes individual or group therapy, guidance for parents and educational teams, local emergencies, psychological assessments and support for special education students [1, 2]. According to the Israel MOE policy as set out in Paragraph 2.2C of Director General Directive 5770/8(a) (“Individual and System Prevention and Intervention Sequences”) [3], educational psychologists are expected to promote prevention by working on both the individual and the system level. To this end, the importance of early identification of developmental needs is emphasized, and in particular risk vulnerability, special needs, life crises and the like. The chances for providing children access to educational-psychological services at critical developmental crossroads, and particularly at the early stages of development (i.e., prevention), depend upon how accessible these services are to children and their families [3]. Yet in the everyday reality of educational psychology in Israel, psychological services for the most part are provided on a limited basis to the population at large, and there are practical difficulties in implementing the principles outlined by the MOE [4, 37]. In some locations, particularly in the peripheral areas, problems of psychologist availability and accessibility are even more pronounced due to difficulties in filling psychologist positions. Consequently, each educational psychologist is responsible for a greater number of educational settings and students than what is mandated by the MOE [5]. These growing burdens together with increasing demands from clients and from the MOE have limited the ability of educational psychologists to provide the best responses to the various challenges to children’s emotional health during routine times and in emergencies. Preference is often given to assessment and bureaucratic duties relating to special education, as well as to individual and national emergencies [37]. Therefore, short-term interventions during routine times that focus on the everyday lives of children and their families are relegated to the margins when determining practical public educational psychology services priorities, thus limiting the prevention and the promotion of emotional well-being [4].

Due to the nature of the educational psychologist’s work as described above, the internet has the potential to help public Educational Psychology Services cope more effectively with the challenges of the twenty-first century and with the complexities of their job. Because we did not know the extent of online public Educational Psychology Services in Israel, we conducted a survey regarding their current online prevalence, with specific focus on the Arab minority and the peripheral regions. To the best of our knowledge, no other such survey has been conducted in Israel to date. Moreover, a database search did not yield information on any similar surveys elsewhere in the world regarding public institutions providing online psychological services in general and online educational psychology services in particular.

## Method

### Procedure

In January–May 2016, we conducted a survey to examine whether public Educational Psychology Service units in Israel have online websites and if so, to investigate their complexity. A website is defined as “a group of World Wide Web pages usually containing hyperlinks to each other and made available online by an individual, company, educational institution, government, or organization” [38]. Therefore, a single page was not considered a “site” in our survey. We sought to examine the extent to which the websites of the public Educational Psychology Services units were accessible to the average end-user seeking information over the internet. We assumed that most of the end-users would be adults – parents and educational staff – who are to some extent aware of the existence of public Educational Psychology Services. Thus, in searching for psychological information or psychological support regarding various difficulties that children encounter, these adults would look for such sites. As far as we know, children do not have this awareness. Still, an end-user can be any client of an educational psychologist, including children [3]. Because Educational Psychology Service units are offered as a public service by the local government authority in which they are located, we conducted a google search that included the name of the local government authority (e.g., city, town, regional council) and the key term “Educational Psychology Service.” The search yielded only the websites of public rather than independent sites of Educational Psychology Services. The survey was conducted in Hebrew and in Arabic.

### Participants

The survey’s participants were in effect all of the public Educational Psychology Service units in Israel. The 252 public Educational Psychology Service units in Israel are divided into six districts. One hundred sixty-nine units are located in cities or localities that are populated mostly by Hebrew speakers. Eighty-three units are located in cities or localities that are populated mostly by Arabic speakers. The vast majority of the units’ internet site (*n* = 45) or internet single page (*n* = 76) were not protected by password, thus allowing people to help themselves by accessing the professional information posted on the internet. Note that maintaining the confidentiality of clients’ personal information is imperative, as required by law [34, 39]. Therefore, sites that offered online support via forums protected those areas with a password. Only one of the units was not accessible at all during the time of the survey because a password was required to access the local government authority’s site, which included the Educational Psychology Services site.

### Measures

We examined several dimensions of the public Educational Psychology Service internet sites:

A. Incidence of public Educational Psychology Service websites:
  1. An Internet site exists: The local Educational Psychology Service unit had a designated site that included more than a single page. When a site existed, the number of active pages was also registered.
  2. No Internet site exists: The local Educational Psychology Service unit was not found via the google search, or the local authority’s website only briefly mentioned the service (usually within the education department’s web page). We differentiated between zero pages (indicative of no notification in the google search we conducted) and one page (which does not qualify as a site but does indicate minimal internet presence).
B. Possibilities for interactive contact with the psychologists working in the unit:
  1. The site is not at all interactive (no possibility for the end-user to contact the psychologists, and no information regarding means of contact).
  2. Low level of interactivity (telephone/email contact details).
  3. High level of full interactivity (via forums) enabling end-users to contact psychologists through the site itself.
C. Type of information provided by public Educational Psychology Service unit website:
  1. Basic information, such as a short description of the unit’s work.
  2. Limited information regarding the unit’s areas of operation and specialization, e.g., treating anxiety, separations, emergencies, entering the first grade and more.
  3. Abundant and diverse information that includes articles and links to other sites.

## Results

### A. Incidence of public Educational Psychology Service websites

Of the 252 Educational Psychology Service units examined, only 17.8% (*n* = 45) have internet sites (i.e., more than one page). For 131 Educational Psychology Service units in Israel, nothing came up in our search, namely 0 pages (52%). Seventy-six units (30.2%) have only a single page, which does not qualify as a website [38]. Thus, 82.2% of all the units surveyed (*n* = 207) have no website at all; 32 units have two to six pages (12.6%); and 13 units have more than ten pages (5.2%). The city of Ramat Gan’s site has the greatest number of pages (*n* = 93) (Ramat Gan public Educational Psychology Service site).

Table 1 shows the prevalence of sites within the Jewish and Arab sectors. Out of 170 units in the Jewish sector, 45 (26.5% of the Jewish units) have more than one page, thus qualifying as an internet site, while none of the units in the Arab sector has a site. Most of the Arab units have zero pages (*n* = 78; 95.1% of the Arab units), and only four units (4.9% of the Arab units) have one page, which does not qualify as a site. Within the Jewish sector the ratio is different, with 53 units (31.2% of the Jewish units) with zero pages, and 72 (42.3% of the Jewish units) with one page.

**Table 1:** Incidence of public Educational Psychology Service websites in Israel, and percentage of websites within the Jewish and Arab sectors

	Jewish Sector	Arab Sector	Total
Internet site exists (more than one page)	45 (26.5%)	0 (0%)	45 (17.8%)
No Internet site exists: 0 pages	53 (31.2%)	78 (95.1%)	131 (52%)
No Internet site exists: 1 page	72 (42.3%)	4 (4.9%)	76 (30.2%)
Total	170 (100%)	82 (100%)	252 units (100%)

Table 2 shows the prevalence of sites within each of the six regions in Israel. The number of units in each region varies. The northern region has the most units (*n* = 90 units; 35.7% of all units), and the Tel Aviv region has the smallest number of units (*n* = 15; 6% of all units). Yet the rate of internet sites is highest in the Tel Aviv region (*n* = 9; 60% of the units in that region), whereas the rate of internet sites is lowest in the northern region (*n* = 6; 6.7% of the units in that region), and in the Haifa region (*n* = 2; 6.7% of the units in that region).

**Table 2:** Incidence of Israeli public Educational Psychology Service websites by region, and percentage of websites within each region

Region	Internet site exists (more than one page)	No Internet site exists: 0 pages	No Internet site exists: 1 page	Total
North	6 (6.7%)	70 (77.7%)	14 (15.6%)	90 (35.7%)
South	7 (17.5%)	19 (47.5%)	14 (35%)	40 (15.9%)
Jerusalem	6 (28.6%)	5 (23.8%)	10 (47.6%)	21 (8.3%)
Tel Aviv	9 (60%)	3 (30%)	3 (30%)	15 (6%)
Haifa	2 (6.7%)	17 (56.7%)	11 (36.7%)	30 (11.9%)
Center	15 (26.8%)	17 (30.3%)	24 (42.9%)	56 (22.2%)
Total	45 (17.8%)	131 (52%)	76 (30.2%)	252 (100%)

Table 3 shows the incidence and percentage of websites of public Educational Psychology Services in Israel with more than ten pages, organized by region. Examination of the 13 sites with ten pages or more showed that the highest rate was in the Tel Aviv region (*n* = 5; 38.5% of the units with 10+ pages), and the lowest in the Haifa (*n* = 0; 0%) and southern (n = 0; 0%) regions. One surprising finding was that the Educational Psychology Services units in the three major cities in Israel, namely Jerusalem, Tel Aviv and Haifa, do not have an internet site at all or have a site with few pages (2–5).

**Table 3:** Incidence and percentage of Israeli public Educational Psychology Service websites that have more than ten pages, by region

Region	Internet site exists (more than one page)	The site has 10 pages or more
North	6	3 (23%)
South	7	0 (0%)
Jerusalem	6	1 (7.7%)
Tel Aviv	9	5 (38.5%)
Haifa	2	0 (0%)
Center	15	4 (30.8%)
Total	45	13 (28.9%)

Note that an end-user who searches for the key words “Educational Psychology Services” without adding a specific name of a local government authority or regional council will reach the site of the Educational Psychology Service that is part of SHEFI. This site offers abundant and diverse information regarding, among other things, various developmental issues, national emergency situations, and special education procedures. The information is presented in two sections – one addressed to educational psychologists and the other to school counselors. Apparently, this site specifically targets professionals (psychologists, counselors and teachers) that work with children, who constitute only part of the Educational Psychology Services clientele [3]. The vast population that uses the services of educational psychologists, namely children and their parents, are not directly addressed on this site.

The Educational Psychology Service websites that were found (*n* = 45) underwent additional examination in order to map the functions they provide to the users.

### B. Possibilities for interactive contact with psychologists

Of the 45 Educational Psychology Service websites, two did not offer the option of interactive contact with psychologists (Level 1) (4.4%); forty sites allowed a low level of interactivity (Level 2) (88.8%); and only three sites (6.7%) offered a high level of interaction (Level 3), all in the Jewish sector, for example the Beit Shean Public Educational Psychology Services site.

### C. Type of information provided by unit website

All the Educational Psychology Services units that had websites offered abundant and diverse information (Level 3). None of these websites provided only a basic level of information (Level 1) or limited information (Level 2).

The information posted on these websites was written mostly for an adult audience, namely parents and educational staff, as reflected in the complexity of the language, the topics and the graphics. In effect, only one website specifically and directly addressed children and adolescents.

## Discussion

Growing numbers of children and adolescents and their parents in Israel and throughout the world have direct access to the internet and spend countless hours online. Public Educational Psychology Services in Israel aimed at supporting child development and enhancing the emotional welfare and mental health of children and their families must operate within this reality. The current survey examined the prevalence and characteristics of the internet sites of all the public Educational Psychology Service units in Israel in the Jewish and Arab sectors.

As described above, of the 252 Educational Psychology Service units examined, only 17.8% (*n* = 45) have internet sites (i.e., more than one page). Of these, 32 units have sites with two to six pages (12.6% of all the units) and 13 units have sites with more than ten pages (5.2% of all the units). The survey indicated that 207 Educational Psychology Service units in Israel (125 in the Jewish sector and 82 in the Arab sector), constituting 82.2% of all the units surveyed, do not have a webpage at all or have only a single page. This state of affairs is in stark contrast with findings in Israel and worldwide pointing to frequent and widespread internet usage among adults, adolescents and children and raises questions regarding the possible causes.

We hypothesize that one explanation may be related to the nature of the work of educational psychologists in Israel, which is marked by a heavy load of diverse tasks and a frequent need to cope with emergencies and crises [1, 2, 4, 36]. These factors are liable to make it difficult to plan and develop new work areas such as creating an internet site. Another possible cause is the training and socialization processes that psychologists undergo, which place emphasis on FTF interaction with clients. Psychologists may therefore perceive the internet media as very different and as diminishing their professional identity as psychologists, thus provoking suspicion and hesitation. In effect, we encountered similar responses from psychologists who participated in various international workshops we conducted ([40], (Alkalay S, Dolev A, Maital SL, Pfohl W: Creating internet-based services: professional frontiers in a digital age, unpublished), (Alkalay S, Dolev A, Maital SL, Pfohl W: Challenges in implementing internet –based school psychology services, unpublished)) and in a recent survey among 78 Israeli educational psychologists (Alkalay S, Dolev A: Can we assist vulnerable children and adolescents via the internet? – Israeli school psychologists’ perceptions and attitudes, unpublished). Despite positive attitudes and recognition of the importance of the internet to their professional work, many psychologists stated that they do not use the internet regularly in their work. Among the reasons mentioned for not using the internet were ethical issues and apprehensions that the internet deprives psychologists of a vital component of their work, namely FTF interaction with the client. Similar findings were reported regarding the responses of UK psychologists on an internet forum [41]. The psychologists’ responses suggested that although participants recognized the great potential inherent in therapy offered online, they also expressed concerns regarding regulation and ethical issues, and recommended that minimal training requirements should be specified.

Complementary research is needed that includes MOE policymakers, the chief educational psychologists and chief regional psychologists within the MOE, and the directors of the public Educational Psychology Services units. Such a study is needed in order to further clarify perceptions regarding assimilation of the internet as a viable professional tool for educational psychologists as well as possible obstacles to this goal.

An interesting finding of our survey is that none of the public Educational Psychology Services units within the Arab sector has a website. Only four units (4.9% of the Arab units) had one page, which does not qualify as a site, while our google search did not find the vast majority of the Arab units (indicative of zero pages) (*n* = 78, 95.1%). Thus, despite the marked advantage in using the internet to boost the psychological services available to this population group, it appears that this media is barely used within the Arab sector.

We speculate that this situation is due partially to the shortage in the resources needed to develop Educational Psychology Services websites in the Arab sector. Specifically, a major shortage of Arab psychologists has been documented. While the Arab minority in Israel constitutes 20.9% of the population, only 1.4% of the clinical psychologists in Israel are Arabs, and only 4% of all M.A. graduates in psychology in Israel are Arabs [42]. Furthermore, the public Educational Psychology Services units in many of the Arab localities are very small, often comprising only one or two psychologists. Thus, many of the Arab Educational Psychology Services units in Israel face a manpower shortage. The chief Educational Psychologist in the MOE reported that within the Arab units, the standard required ratio of approximately one psychologist per 1000 children is not achieved. Most of the units in the Arab sector met only 43% of the mandated ratio in 2010, and in 2017 the situation improved, but the units still met only 63% of the ratio [7]. Additionally, most of the Arab population lives in the peripheral northern and southern regions, where the number of mental health professionals and mental health services in general is deficient. Furthermore, alongside the growing demand for psychological services, the public Educational Psychology Service presents a viable, less stigmatized and more accessible solution for the Arab population compared to other mental health services [42]. These conditions have created a work overload for the Arab Educational Psychology Services. Under such circumstances it is very difficult to allocate human and fiscal resources to developing and maintaining a website.

In addition to the shortage in manpower, the Arab units also experience the same fiscal deficiencies that characterize the Arab sector in general. For example, a governmental investigative committee in 2000 known as the Or Commission found that only 69.4% of funds designated for investment in the Arab localities (in education, welfare, infrastructure etc.) were in fact transferred to the local authorities. A follow-up report found that three years later Arab localities still received lower governmental funding compared to Jewish localities [43]. This marked insufficiency in financial resources may present an additional obstacle to developing websites for the public Arab Educational Psychology Services.

A complementary possible explanation for the relative lack of online presence of the Arab Educational Psychology Services may be related to the general pattern of health-promoting behaviors among the Arab population of Israel [44]. Compared to Israeli Jews, the Arab population in Israel has apparently not yet widely adopted health-promoting behaviors. For example, only 30% of the adult Arab population regularly engages in physical activity, compared to 52% of the Jewish population. When it comes to promoting mental health, a matching pattern is revealed. Thus, adult Arabs who are in emotional distress are less prone than adult Jews to seek help from mental health professionals (21 and 39%, respectively) [44]. A similar discrepancy exists between Arab and Jewish adolescents with respect to seeking help from mental health professionals. In an Israeli study of a large adolescent sample, out of those who met the criteria for mental disorder (10–12%) [23], 91% of the Arabs vs. 54% of the Jews did not seek help from any educational or mental health professional [24]. These findings may reflect reluctance among the Arab minority to use mental health services due to stigma [42], similar to Khan's findings (Khan MA: Exploring black, Asian and minority ethnic young people’s attitudes towards accessing online and face-to-face counselling, unpublished) regarding minorities in the UK.

Our results pointing to a discrepancy between the Jewish and Arab sectors in Israel regarding the pattern of prevalence of public Educational Psychology Services websites are in accordance with a broader trend seen in Israel. Likewise, a survey of 34 internet sites of Israeli government units revealed that a third of the sites did not have any information posted in Arabic, and on more than a third of the sites the information posted in Arabic was not as extensive, updated, and high quality as the Hebrew information [45].

Another interesting finding of our survey refers to the pattern of prevalence of internet sites in the various regions in Israel. Examination of the distribution of the sites across the six regions in Israel reveals an interesting picture. The Tel Aviv region has the smallest number of Educational Psychology Services units (*n* = 15), constituting only 6% of all the units in Israel. Yet this region has 1,388,400 residents who reside in a few relatively large urban localities [25]. The city of Tel Aviv is one of the three largest cities in Israel and is considered the central metropolis. In fact, the Israeli Peripherality Index of Localities and Local Authorities is calculated based on distance from the Tel Aviv regional border [46]. Our data indicate that 60% of the public Educational Services in the Tel Aviv region have internet sites (*n* = 9), constituting the largest proportion of sites compared to other regions. Furthermore, we found that five units in the Tel Aviv region have sites with ten pages or more, constituting 38.5% of large sites of that type. In fact, the site with the largest number of pages was developed by one of the cities within the Tel Aviv region. The findings regarding the peripheral regions reveal a different picture. The northern region has the largest number of Educational Psychology Services units (*n* = 90), constituting 35.5% of all the units in Israel. This region has almost the same number of residents as in the Tel Aviv region – 1,401,300 – living in 419 relatively small localities and a few large cities [25]. Our data indicate that only 6.7% of the units in the northern region have internet sites (*n* = 6). It is interesting to note that three units in the northern region have sites with ten pages or more, constituting 23% of large sites of that type. Examination of those sites revealed that one of them belonged to a regional council municipality with relatively high socioeconomic status and the other two sites belonged to cities in outlying areas that received extensive digital support via a privately-owned fund (A Password for Every Student project—*Sisma Lechol Talmid*). Forty Educational Psychological Services units operate in the southern region, constituting 15.9% of all the units. This peripheral region also has almost the same number of residents as the Tel Aviv region – 1,244,200 – living in 252 relatively small localities and a few large cities [25]. Our data indicate that 17.5% of the units in the southern region have internet site (*n* = 7). Yet none of the units in the southern region has a site with ten pages or more. Both of the peripheral regions have the largest number of localities with a high peripherality index [46].

After integrating all the data, we reached the conclusion that it is more likely for an Educational Psychology Service unit in the large central cities to have an internet site, while the peripheral regions have fewer such sites. Note that the population of the peripheral regions includes a high proportion of Arabs [47]. This is reflected in the ratio of Arab units in each region and especially when comparing the northern region - 63.3% Arab units (*n* = 54) - with the Tel Aviv region, which has no Arab units at all. Thus, the data may contain a confounding factor, which as described above also contributes to the relative scarcity of internet sites in the peripheral regions. It is also apparent that establishing an internet site may be facilitated by privately owned funds. Moreover, it seems that the mere size of a city or its proximity to the Tel Aviv metropolitan center is not sufficient for establishing a site or for developing a more diverse site, as is evident from the fact that Israel’s three major cities – Jerusalem, Tel Aviv, Haifa – either do not have internet sites at all or have very limited sites consisting of three to five pages.

Of the 45 public Educational Psychology Service units that had websites, most were not at all interactive (*n* = 2; 4.4%) or had a low level of interactivity (*n* = 40; 88.8%). A possible explanation may stem from the natural fear of changes of any sort. Fox listed a number of reasons for fearing and resisting changes, one of which is apprehension of the unknown and unfamiliar leading to fear of losing control [48]. Interactive communication can be perceived as unknown and unfamiliar (e.g., receiving messages from clients at unexpected hours), and this perception may explain the greater preference for creating non-interactive websites. Furthermore, for many psychologists, the internet is a new and foreign land when it comes to professional issues. Many of the psychologists are not “natives” to this digital arena and thus may feel incompetent in providing services online.

In addition to calling attention to the problems involved in embracing the internet as a means of psychological support, our examination of the 45 existing public Educational Psychology Services internet sites also reveals an optimistic and encouraging perspective. All of these websites posted abundant and diverse information. This kind of information provides a convenient and helpful form of self-help, enabling adults and children alike to visit the websites and read professional materials that can assist them in coping with adversities. In fact, this way of sharing psychological knowledge is in line with George A. Miller’s well-known call in his APA (American Psychological Association) presidential address to “give psychology away” [49].

The tremendous amount of practical psychological information possessed by public Educational Psychology Services today can be made available to the entire population, thus providing plentiful options for preventing and moderating common life crises as well as for contributing to additional mental health aspects. We therefore believe that the active presence of public Educational Psychology Services on the internet will facilitate access to psychologists and enable the dissemination of professional and reliable information to the public. This viewpoint is in line with the code of ethics of psychologists in Israel, and particularly with the principle of social responsibility. According to this working principle, “psychologists must be aware of their professional and scientific responsibility to the community and the society in which they work and live. They must implement and inform the public of their knowledge of psychology with the goal of contributing to human well-being” [50].

We found it interesting that the information posted on the websites was mostly written for adult audiences, namely parents and educational staff, in terms of complexity of the language, the topics, and the graphics. In effect, we discovered only one website that directly appealed to children and teens and offered them age-appropriate psychological information regarding areas that affected them, such as social problems or family arguments. This finding may reflect the perceptions of educational psychologists regarding their clients and their own professional role. Many educational psychologists in Israel dedicate a significant amount of their working hours to promoting children’s well-being via working with the child’s significant adults (parents and educational staff) (e.g., [51]). Thus, it is understandable that the Educational Psychology Services internet sites post information that targets the adult population. However, we believe that it is imperative for Educational Psychology Services internet sites to address children and adolescents as well. Children will search for information relevant to them on the internet even if it is not supplied by a reliable professional source. Bozkurt et al. [52] found that in a normative sample of children and adolescents (aged 8–17), 69.7% used the internet to search for information. We believe that information provided by the public Educational Psychology Service has the advantage of being reliable and professionally responsible. Because the Educational Psychology Service is a professional, public and supervised body, the information it disseminates conveys to the public an impression of “quality assurance by the MOE.” Thus, children and teens searching for psychological knowledge about various problems would benefit if the public Educational Psychology Services’ sites were to address them specifically and offer relevant information in a comprehensible and age-appropriate manner.

To our understanding, expanding the operational channels of educational psychologists to the internet is congruent with Israeli policy governing electronic media, as published in 1997 in the State of Israel’s report on Preparations for the Digital Era [45]. This report states that all government and local authority units are expected to establish internet sites. The sites are to provide the citizens with updated information, thus enabling people to utilize their rights to the fullest. Indeed, medical institutions in Israel have already begun using telehealth. Telehealth services facilitate personal, tailored care at current professional standards and rapid response to patients via digital means (e.g., [53]). For example, in 2012 Maccabi Healthcare Services in Israel founded Maccabi Telecare Center (MTC), a multidisciplinary healthcare service providing telemedical care to patients with complex chronic conditions [54]. We believe that expanding the educational psychology services to include online services is in line with current approaches to public services in general, and specifically in providing medical and mental health care to clients. Providing educational psychology services via the internet is also in accordance with the strategic trend in educational psychology, as presented by the Israel MOE’s chief psychologist [55]. Friedman described an intra-organizational process of clarifying the organization’s goals for the twenty-first century. Six district focus groups were held in 2015 among educational psychologists with varying professional status and experience, as well as a focus group for educational psychology interns in cooperation with the interns committee. According to Friedman [55], one of the issues raised in all the focus groups was “the development of educational psychology vis-à-vis the community.” The psychologists in the focus groups raised the need to reify educational psychology with respect to its salutogenic aspects, for until now educational psychology in Israel has mainly been identified with difficulties and developmental impairments along with emotional and interpersonal crises. The focus groups underscored the need to initiate diverse educational psychology activities directed at providing the public with widespread knowledge regarding a variety of developmental, normative and psychological issues throughout childhood and adolescence. We believe that this process reflects the widespread approach of many educational psychologists who advocate activities geared toward prevention more than those geared at intervention.

Thus, in principle there is organizational support for implementing the internet as a media for expanding psychological services. Individual educational psychologists (not as organizational representatives) have also expressed opinions advocating the internet as part of the psychological services. At international and Israeli workshops led by us, as well as in a survey we conducted, we asked psychologists to what extent they believe the internet will be an integral part of the work of educational psychologists in another five years. Most answered that in the future they will use the internet on a daily basis in their work as educational psychologists and cited its advantages. For example, they mentioned the opportunity provided by the internet to meet with children in their natural environment, to disseminate psychological knowledge, to help people who find it difficult to attend therapy sessions overcome their initial hesitation, and to surmount problems of limited access due to distance or physical restrictions. The advantages of the internet for the psychologists themselves also came up: access to up-to-date information, training options, possibilities for creating strong professional networks that provide backup and support as well as access to experts in specific fields of knowledge ([40], (Alkalay S, Dolev A, Maital SL, Pfohl W: Creating internet-based services: professional frontiers in a digital age, unpublished), (Alkalay S, Dolev A, Maital SL, Pfohl W: Challenges in implementing internet–based school psychology services, unpublished), (Alkalay S, Dolev A: Can we assist vulnerable children and adolescents via the internet? – Israeli school psychologists’ perceptions and attitudes, unpublished)).

In examining the results of our website survey, we identified encouraging trends regarding implementing the internet as a medium for educational psychology work. Particularly encouraging is the finding that all of the public Educational Psychology Service units with websites (*n* = 45) provided a wealth of information. Of those sites, 28.8% (*n* = 13) comprised more than ten pages. To our understanding these findings indicate that the Israeli public Educational Psychology Services units that dared to take the initial leap into the digital arena wish to share their vast knowledge with the community and understand that this is an effective media for psychological support. It is our hope that this trend will grow, leading to a constant increase in the number of public Educational Psychology Service units that join the digital world and to an abundance of psychological knowledge accessible to the community.

### Limitation of the research

A possible explanation for the lack of internet sites within the Jewish sector may be related to level of religious observance. The ultra-Orthodox population (Haredim) constitutes approximately 14% of Israel’s Jewish population [56]. One of the main characteristics of Haredim, is isolation from the general population as expressed by independent education systems, special dressing codes, etc. [57]. This characteristic may foster a perception of Haredim as abstaining from the internet. Thus, a large Haredi population in a locality can influence the local public Educational Psychology Service unit in deciding whether to develop and operate an internet site. Since the percentage of Haredim in each locality is unknown, we were unable to investigate this assumption. Further research is needed in order to clarify this issue.

However, a rapid and stable trend of rising computer and internet usage among Haredim has been evident since 2008 [58]), and according to current survey data, around 50% of Haredim over the age of 20 use the internet [58–60]. Although information is lacking regarding internet use among children and adolescents in the Haredi sector, it can be assumed that they are also exposed to computers and the internet in their immediate surroundings. Thus, the Haredim are also potential candidates for consuming online educational psychology services. The most popular websites among Haredi users are news sites and religious contents sites, which are characterized by purposefulness and focus on content. Moreover, Haredim use social networks much less than the general public in Israel [57]. Consequently, SHEFI should take the unique cultural sensitivity of the Haredi community into consideration in designing suitable websites.

Also, as previously mentioned, we do not know what encourages or hinders the director of an Educational Psychology Service unit to develop an internet site. In the absence of an explicit and clear general policy regarding unit internet sites, each director has autonomy in allocating the unit’s resources. Thus, directors’ attitudes regarding establishing a site along with the resources at their disposal constitute a vital component in the decision regarding whether to set up an internet site. We did not investigate these attitudes in our research and we suggest future research in this direction.

Looking beyond policy recommendations, we believe that the MOE via SHEFI should consider taking practical action by instituting a central computer unit to be responsible for all technological necessities in producing and supporting the websites of the local units. Thus, a local Educational Psychology Service unit that wishes to join the array of already existing websites could easily do so by using this central computer unit.

## Conclusion

Today’s technological reality should be taken into consideration on the road to achieving the main target of the Educational Psychology Services, namely supporting children and enhancing their welfare. In light of endless efforts to meet the demands faced by the public services, the work overload, and the shortage in manpower, technology can offer a way to overcome these obstacles. In the reality of 2019, it is customary and expected to receive services online. It thus appears that public Educational Psychology Services in Israel should evolve professionally and embrace technology. We believe that the internet is likely to serve as a significant means of expanding the mental health services for children and youth in Israel, making them accessible to the entire population.

The government of Israel has encouraged this trend by publishing an official policy directive concerning the digitation process in the State of Israel [45]. However, to date there is no explicit policy regarding offering online Educational Psychology Services or other online mental health services for children and youth. Additionally, no funds provided by the government or the local authorities have been designated to promote development of public Educational Psychology Service websites. This may present an obstacle for all the public services, but especially for the units which operate in the peripheral regions and for units in the Arab sector, which operate under more challenging conditions compared to the Jewish sector. In order to promote the development of Arab Educational Psychology sites, we suggest translation of the existing Hebrew sites as a possible initial move. This is similar to the policy adopted by the internet sites of government units [45].

The emerging picture of the online psychological services in Israel has promising aspects as well. Although the majority of the public Educational Psychology Services units do not have websites, the 45 existing public Educational Psychology Services internet sites posted rich and diverse information. Websites were more prevalent in the Tel Aviv central region, and this region had a higher percentage of sites offering rich and extensive information compared to the peripheral northern and southern regions. Nevertheless, we also discovered that the size and centrality of a locality are not sufficient for establishing a site. Some small and peripheral localities also had well developed sites. Yet such an endeavor may be more challenging for peripheral and Arab units, which operate under more complex conditions. Thus, we call for policymakers of mental health of children and adolescents to adopt the internet as a means for overcoming the population’s unmet needs for emotional support and mental health services for children and youth and to expedite an explicit and funded call for action. We believe that a joint effort of the MOH, MOSAS, and MOE (via SHEFI), will facilitate this progressive process and the implementation of internet-based mental health services for children and youth in Israel. We propose that SHEFI and the educational psychological services could provide a case-study for developing joint technological infrastructures in collaboration between the ministries. Those infrastructures should be adapted to the unique characteristics of the educational psychologists’ work - namely, promoting the emotional well-being of children and youth in the population at large, and doing so while working in schools.

Ethical issues should be considered in formulating such a policy, and in establishing and operating websites in the public Educational Psychology Services. Additionally, the internet sites should take cultural diversity into account (e.g. by considering the special characteristics of the ultra-orthodox population and the Arab sector) by posting professional information that is culturally sensitive. Finally, the future presence of SHEFI on the web should also consider possible obstacles and limitations of internet use that might have negative effects on some users. Thus, we recommend that the process of establishing internet sites for the public Educational Psychology Services should be accompanied by the Ethics Committees of SHEFI and of the Israeli Psychological Association.

## Abbreviations

APA: American Psychological Association
CAMH: Child and Adolescent Mental Health
CBT: Cognitive Behavioral Therapy
FTF: Face to Face
MOE: Ministry of Education
MOH: Ministry of Health
MOSAS: Ministry of Social Affairs and Services
MTC: Maccabi Telecare Center
PTSD: Post Traumatic Stress Disorder
SHEFI: Sherut Psychology Yeutzi (Hebrew) - The Psychological and Counseling Services Division of the Israeli Ministry of Education
